# Mapping the knowledge structure of frailty in journal articles by text network analysis

**DOI:** 10.1371/journal.pone.0196104

**Published:** 2018-04-19

**Authors:** Youngji Kim, Soong-nang Jang

**Affiliations:** 1 Department of Nursing, College of Nursing, Gachon University, Incheon, Republic of Korea; 2 Red Cross College of Nursing, Chung-Ang University, Seoul, Republic of Korea; Taipei Veterans General Hospital, TAIWAN

## Abstract

**Backgrounds:**

This study was to understand the trends of frailty research and networking features of keywords from the academic articles focusing on frailty in the last four decades.

**Method:**

Keywords were extracted from articles (n = 6,424) retrieved from Web of Science, from 1981 to April 2016, using Bibexcel, and a social network analysis was conducted using Net Miner.

**Results:**

The core-keywords of research on frailty are constantly changing over the last 40 years. The keywords were tended to focus on impact in the 1980s, and moved to the determinants (i.e., malnutrition) in the 1990s and the 2000s, and in the 2010s, most of keywords were about determinants and measurement of frailty. In the early stages of frailty research, individual behaviour modifications were emphasized as intervention. Keywords with the highest degree centralities were ‘impact’ (1980s), ‘frailty’ (1990s), ‘home care’ (2000s), and ‘dementia’ (2010s). Keywords with the highest betweenness centralities were ‘model’ (1980s), ‘frailty’ (1990s), ‘chronic disease’ (2000s), and ‘malnutrition’ (2010s).

**Discussion:**

This study provides a systematic overview of frailty knowledge development. ‘Dementia’ was found to be the keyword with the highest degree centrality, showing that studies on cognitive function are those being most actively conducted in recent decade. In the 2000s frailty research, sub-themes were sarcopenia, dementia and disability, indicating that frailty was investigated from the view of disease. In the 2010s, obesity, nutrition, prevention, evaluation, and ADL (activities of daily living) were sub-themes of the research network that focused on frailty prevention.

## Introduction

Frailty has become an important issue with the increase in the number of frail elderly as a result of the growing elderly population. The term frailty was first used to indicate elderly individuals, above the age of 75, who need help to maintain their daily lives, have a physical disability, emotional damage, and/or inadequate physical/social environment [1]. Frailty can be observed in all age groups, but its importance is especially emphasized among the older people. This is because frailty is identified as a factor that predicts negative outcomes, such as an increased risk of disability, falls, and hospitalization [2, 3]. Moreover, frailty is known to be the intermediate stage between a healthy state and disability, in which disability can be delayed or prevented, through appropriate interventions. Older people who have already developed disability show limitations in independent activities, such as self-care. They also have higher tendencies to rely on long-term care insurance, which can exacerbate care burdens. Therefore, there is a rising need for the early detection of frailty, which can enhance the maintenance of daily life activities of the elderly, and prevent disabilities, before this population enters long-term care.

Even the frailty is widely investigated in gerontology and other fields, concerns have been raised that the concept is used in multiple ways, depending on the perspective and understanding of the researcher, without a clear definition [4]. There are no generally accepted instruments that measure frailty, because a definition of frailty has not yet fully established [5, 6]. In previous studies, measurements for frailty have focused on the physical aspects or phenotype of the condition, while only some instruments have addressed psychological aspects [7]. Frailty is gradually becoming recognized as an incorporated concept with multidimensional features, including physical, mental, social, and environmental factors [8].

In order to better understand frailty, it is necessary to review the main topics that have been reported in previous studies. Fortunately, there are a large amount of accumulated data, as frailty-related studies have been extensively conducted since the 1980s. Meta-analyses and systematic reviews as a method to comprehensively analyse previous research have been also conducted. However, meta-analysis and systematic reviews has some limitations, such as it applies a strict methodology, and it is incapable of covering the entire research area [9–11]. It has become increasingly difficult to accommodate the entirety of research data through use of existing literature review methods.

Social network analysis (SNA) has been utilized by many fields of study to analyse vast amounts of data, examining knowledge structures, interaction processes, and relationships [12]. SNA is a method that has the strength of being able to cover an entire research area, since it targets data accumulated from the beginning of a particular research topic to the present [13]. Text network analysis, which investigates research topics of related literature by social network analysis, has been adopted in various academic disciplines [14].

One of the biggest differences between the conventional literature analysis and text network analysis is that, while the former focuses on qualitative analysis of data generated in previous studies, the latter quantitatively explores the knowledge structure in the research area. Network analysis utilizes defined pairs of words (morphemes) existing next to each other as co-occurrences in literature, and provides ample information by extracting and visualizing the network of the words’ semantic relationship [15].

Contents analysis takes time and labor for collection and classification of materials with a qualitative approach, and must rely heavily on experts’ knowledge, experience, and insights to perform the analysis [16]. Using key concepts and frequency of appearance of predefined classification themes, we can learn how to treat specific topics exactly; however, there is a limit to understanding the relationship and structure of themes. It is difficult to determine the meaning of a certain word by counting how often it appears in a text. Frequently occurring words occupy a lot of space in the main text. Our interest is not in one word that is often displayed, but rather in words that will be the focus of discussion. The most significant advantage of text network analysis is that it helps understand what a text primarily discusses practically and potentially by looking into the links between the words used in it. Network-based review has sufficient advantages as a complementary measure to traditional analysis methods.

This study aimed to identify core keywords of frailty research using text network analysis, understand the trends of frailty research and networking features of keywords from the academic articles focusing on frailty in the last four decades.

## Methods

### Data

Journal articles, published from Jan. 1981 to Apr. 2016, were retrieved using the search engine Web of Science. The keywords searched were ‘frail’ or ‘frailty’, and the journal categories were limited to ‘geriatrics’, ‘geriatrics gerontology’, ‘general internal medicine’, ‘public environmental occupational medicine’, ‘nursing’, and ‘psychology’. Only articles written by English were included in the analysis. The search results retrieved 10,367 articles. From these, a database was constructed in 10-year intervals. Two researchers reviewed the retrieved articles according to the criteria for selection and exclusion of data. Specifically, after assessing titles and abstracts of the articles, repeated or irrelevant articles were excluded. A third researcher reviewed the articles for which a consensus was not made. A total of 6,424 studies were selected as the final subjects of analyses.

### Analysis

We performed a SNA to identify knowledge structure of frailty research and to analyse the roles, properties, and rank of keywords using the relationship established between main keywords. Using the Bibexcel program, keywords were extracted from the selected articles, and the extracted keywords were standardized. Bibexcel is a great tool for data mining for bibliographic research [17]. The process of standardization involved removing synonyms or derivatives, and excluding supplementary expressions or words that are irrelevant to the content.

When extracting keywords, general verbs and nouns not related to the content (e.g., research, group) as well as general terms that are rarely seen as key research concepts (e.g., special symbols, additional vocabulary, vocabulary with no direct influence) were removed [18]. As for the inclusion of similar words such as ‘old’, ‘elderly’, and ‘aged’, a professor of nursing, a professor of medicine, and a student of the doctoral course selected keywords that they agreed upon. To examine what the keywords truly meant in texts, the source papers were checked, in an attempt to exclude subjective ideas of research authors. Keywords that the authors of each article suggested were used as analysis unit in this study and in case of keywords such as ‘nursing-home’ that the author selected, were not separated into two words nursing and home, but were processed as one keyword to retain the semantic morphemes. This was done to fully reflect the authors’ intention and investigate the keywords semantically.

Net Miner version 4.0 [19] was used for analysing the relationship between keywords. For text network analysis, building co-occurrence matrix should be preceded. The Net Miner program was used to convert the 2-mode network of the article-keyword structure to 1-mode network of the keyword-keyword structure. This feature is available in NetMiner software.

We used Net Miner to calculate the frequency of co-occurrence of keywords and we generated keyword–keyword matrix by use of the frequency of co-occurrence as weight values. We quantitatively analysed the properties, strength, and correlation between the keywords by calculating Jaccard coefficient [20].

Degree centrality, betweenness centrality and cluster analyses were used to find the important nodes within the network [21, 22]. Degree centrality indicates how many links the nodes have in a network, and it is measured by examining the co-occurrence of a keyword and another one. Degree centrality is at the center of the network, which is regarded an important research theme. Meanwhile, betweenness centrality is measuring ‘how well a node acts a medium or bridge in constructing a network with other nodes’. A keyword with greater betweenness centrality can play an important role in facilitating connective flows between keywords in a network, thereby helping the entire network run seamlessly. Betweenness centrality is useful for identifying the central concept in the research [23]. As keywords with higher betweenness centrality can provide research-related variables, they were chosen to be analysed in this study. Cluster analysis was performed to suggest subgroups of research by time period. Links with the highest betweenness centrality were eliminated using betweenness centrality, while communities were designated based on cohesion, and keywords in each group were listed in the order of connection strength. The community algorithm based on Link Betweenness (which is known as the GN algorithm) was suggested by Girvan and Newman [24]. This algorithm computes the betweenness centrality of all links in the one-mode network, and identifies and removes links with the maximum betweenness value, after which the betweenness of all the links is recalculated. This procedure is repeated until no links remained. As more links were removed, more components appeared, and these components corresponded to sub-groups each level. Finally, keywords with high co-occurrence that were mutually linked in the sociogram and dendrogram were determined as the domain name [25]. The dendrogram is a visual representation of the compound correlation data. The individual compounds were arranged along the bottom of the dendrogram and referred to as leaf nodes. Compound clusters were formed by joining individual compounds or existing compound clusters with the join point referred to as a node.

## Results

### Number of articles and number of keywords

The number of articles was 142 in the 1980s, 753 in the 1990s, 2,643 in the 2000s, and 2,886 in the 2010s, demonstrating a sharp increase from the year 2000. The number of extracted keywords was 62 in the 1980s, 361 in the 1990s, 997 in the 2000s, and 965 in the 2010s (S1 Table).

The density of each network was shown as 0.043 in the 1980s, 0.060 in the 1990s, 0.086 in the 2000s, and 0.090 in the 2010s. The highest ranked keywords were ‘living’ in the 1980s, ‘depression’ in the 1990s, ‘frail elderly’ in the 2000s, and ‘frailty’ in the 2010s.

**Fig 1:**
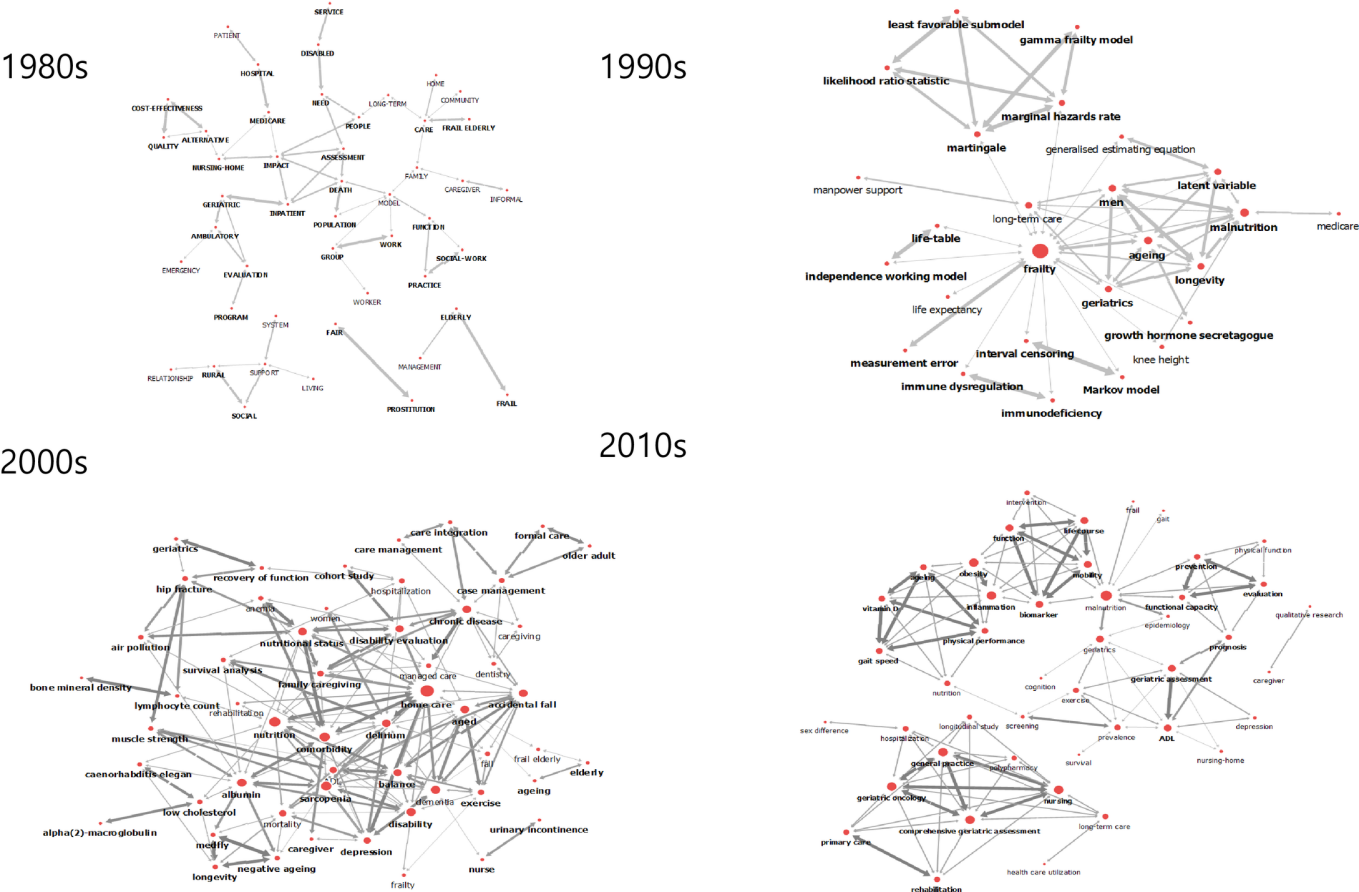
Degree centrality trend through 40 decades.

Analysing the degree centrality showed that the mean degree centrality in the 1980s was 0.07, and the network centralization index was 31.2%. The degree centralities of the keywords were in the order of ‘impact’ (0.17), ‘model’ (0.17), ‘death’ (0.14), ‘care’ (0.14), and ‘assessment’ (0.11). The mean degree centrality in the 1990s was 0.15, and the network centralization index was 64.42%. The degree centralities of the keywords were in the order of ‘frailty’ (0.75), ‘malnutrition’ (0.36), ‘aging’ (0.32), ‘men’ (0.29), and ‘geriatrics’ (0.25). The mean degree centrality in the 2000s was 0.12, and the network centralization index was 25.18%. The degree centralities of the keywords were in the order of ‘home care’ (0.36), ‘nutrition’ (0.30), ‘comorbidity’ (0.26), ‘sarcopenia’ (0.26), and ‘albumin’ (0.23). The mean degree centrality in the 2010s was 0.451, and the network centralization index was 35.98%. The degree centralities of the keywords were in the order of ‘dementia’ (0.79), ‘cognitive impairment’ (0.75), ‘depression’ (0.71), ‘geriatric assessment’ (0.64), and ‘disability’ (0.64; Table 1, Fig 1)

**Table 1:** The Top 15 keywords by centrality according to time period.

Rank	1980s		1990s		2000s		2010s	
	Degree Centrality	Betweenness Centrality	Degree Centrality	Betweenness Centrality	Degree Centrality	Betweenness Centrality	Degree Centrality	Betweenness Centrality
1	impact	model	frailty	frailty	home care	chronic disease	malnutrition	malnutrition
2	model	impact	malnutrition	malnutrition	nutrition	home care	geriatric oncology	geriatrics
3	death	death	aging	long-term care	comorbidity	nutrition	nursing	prevalence
4	care	inpatient	men	medicare	sarcopenia	aging	general practice	screening
5	assessment	geriatric	geriatrics	aging	albumin	accidental fall	obesity	nutrition
6	inpatient	family	longevity	latent variable	dementia	nutritional status	comprehensive geriatric assessment	obesity
7	need	care	latent variable	men	disability	dementia	inflammation	inflammation
8	medicare	nursing-home	long-term care	geriatrics	nutritional status	albumin	life course	ADL
9	ambulatory	people	marginal hazards rate	longevity	delirium	case management	function	geriatric assessment
10	function	long-term	growth hormone secretagogue	growth hormone secretagogue	chronic disease	comorbidity	mobility	prognosis
11	nursing-home	need	gamma frailty model	gamma frailty model	accidental fall	disability evaluation	ADL	life course
12	people	medicare	immune dysregulation	functional movement	aging	disability	geriatrics	function
13	evaluation	function	immunodeficiency	functional status	disability evaluation	hip fracture	biomarker	mobility
14	geriatric	alternative	independence working model	geriatric assessment	balance	sarcopenia	geriatric assessment	biomarker
15	alternative	assessment	knee height	gene targeting	depression	hospitalization	gait speed	exercise

**Fig 2:**
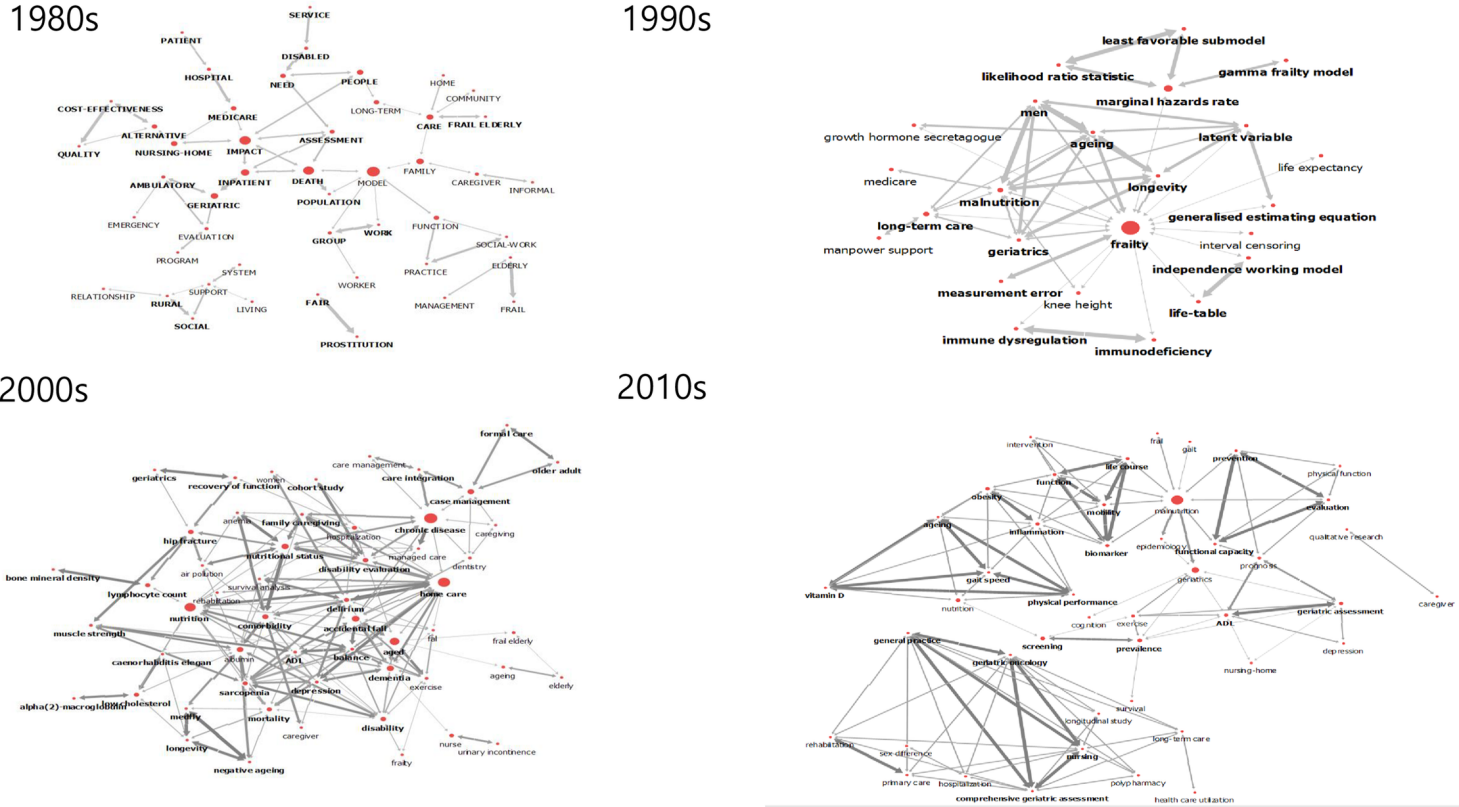
Betweenness centrality trend through 40 decades.

Analysing the betweenness centrality showed that the mean betweenness centrality in the 1980s was 0.09, and the network centralization index was 35.33%. The betweenness centralities of the keywords were in the order of ‘model’ (0.44), ‘impact’ (0.39), ‘death’ (0.30), ‘inpatient’ (0.25), and ‘geriatric’ (0.21). The mean betweenness centrality in the 1990s was 0.04, and the network centralization index was 76.24%. The betweenness centralities of the keywords were in the order of ‘frailty’ (0.28), ‘malnutrition’ (0.06), ‘long-term care’ (0.03), ‘Medicare’ (0.03), and ‘aging’ (0.007). The mean betweenness centrality in the 2000s was 0.03, and the network centralization index was 16.89%. The betweenness centralities of the keywords were in the order of ‘chronic disease’ (0.20), ‘home care’ (0.19), ‘nutrition’ (0.16), ‘aging’ (0.13), and ‘accidental fall’ (0.09). The mean betweenness centrality in the 2010s was 0.00, and the network centralization index was 7.29%. The betweenness centralities of the keywords were in the order of ‘malnutrition’ (0.08), ‘geriatrics’ (0.04), ‘prevalence’ (0.02), ‘screening’ (0.02), and ‘nutrition’ (0.02; Table 1, Fig 2).

**Fig 3:**
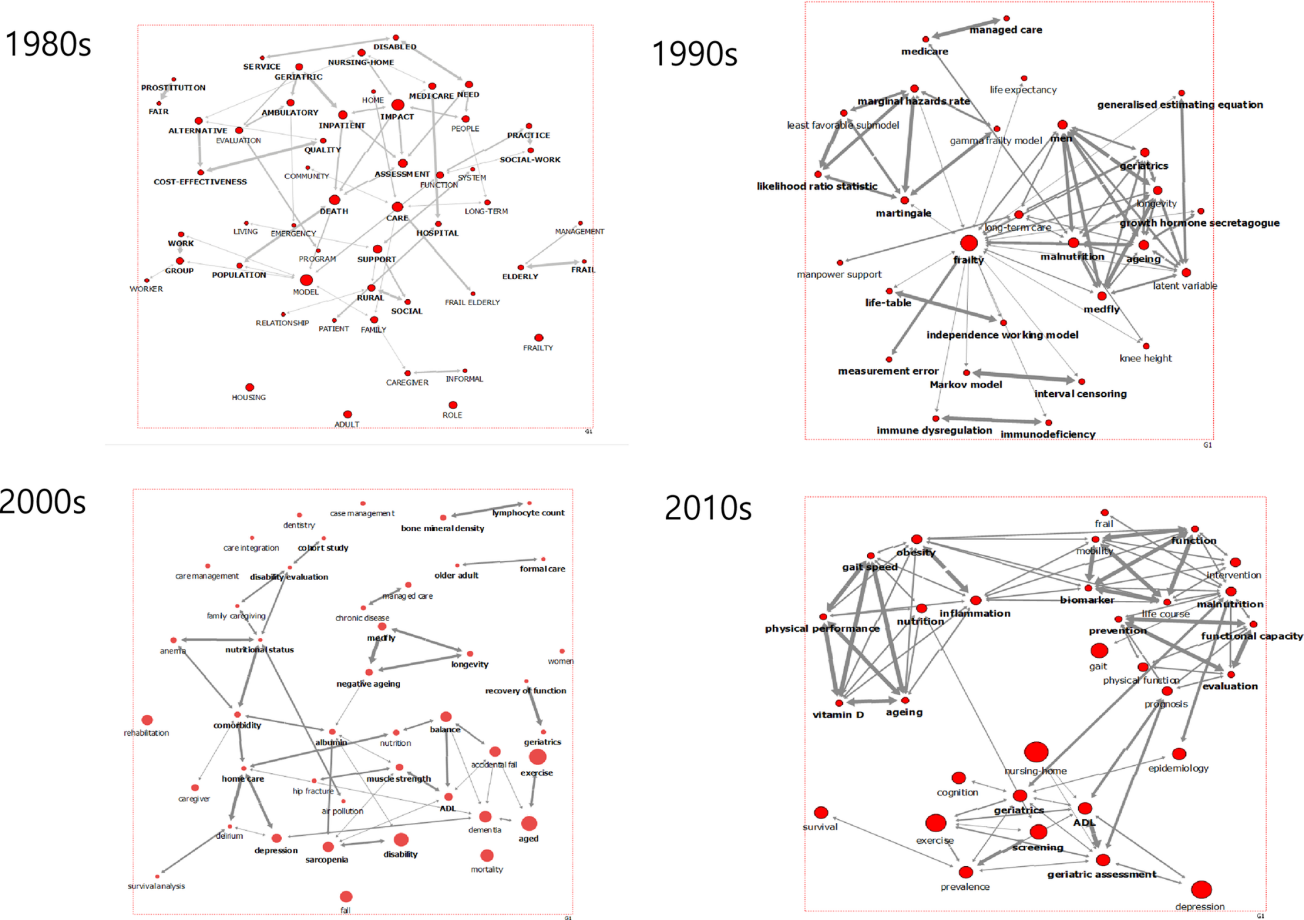
Communities through 40 decades.

Cluster analysis in the network of the research period revealed that the modularity values of the 2000s and 2010s network were 8.41 and 8.42, respectively, which were both more than the standard values of 3.5 points [19], and clustering communities were explored. Table 2 shows the clustering communities. Using the sociogram and dendrogram by group, we read the context in which these keywords were used in articles and named groups of research themes. The results revealed that the main pairs were (community, family) and (nursing–home, women, older) in the 1980s. In the 1990s, (frailty, long-term care, malnutrition), (Medicare, managed care), and (gamma frailty model, marginal hazard rate) appeared in pairs. Among the clustering networks in the 2000s and 2010s, the sub-theme of the network of the 2000s was named (sarcopenia, disability) and (homecare, depression, comorbidity) and the sub-theme of the 2010s was (obesity, nutrition), (evaluation, prevention) and (ADL, geriatric assessment) (Fig 3 and S1 Fig).

**Table 2:** Sub-topic groups of frailty research by cluster analysis.

Period	1980s	1990s	2000s	2010s
**No of clusters**	Undefined	3	16	12
**Modularity**	undefined	2.89	8.41	8.43
**Group 1**	**Community, family**, management, quality, assessment, alternative, approach	**Long-term care, frailty,** aging, **malnutrition,** geriatrics, knee height	**Disability, sarcopenia,** albumin, hip fracture, muscle strength	Gait, inflammation, ageing, **obesity,** mobility, **malnutrition**
**Group 2**	**Older, women**, **nursing-home**, living	**Medicare, managed care**	Aged, exercise, dementia, **depression, home care,** delirium, survival analysis, accidental fall, ADL, balance	**evaluation, prevention,** Functional capacity, physical function,
**Group 3**	Center, provider	**Marginal hazard ratio, gamma frailty model** likelihood ratio statistics, least favorable submodel,	**comorbidity, nutritional status,** Family caregiving, Disability evaluation, cohort study, air pollution, anemia,	**ADL, geriatric assessment,** Prevalence, exercise, geriatrics, nursing-home, survival, cognition

ADL (activities of daily living)

## Discussion

This study aimed to investigate the core-keywords of frailty studies from 1981 to 2016 through text network analysis, examine research trend in each 10-year period, and provide an integrated perspective on frailty research.

In the early period of frailty research in 1980s, ‘impact’, ‘model’, ‘death’, and ‘care’ were found to be the core keywords with high degree centrality. These keywords are belong to main categories related outcomes of frailty rather than understanding of the concept of frailty [26]. Likewise, there are not many studies on determinants of frailty except simple discussions of patterns. As the quantity of research increased, the number of keywords was also increased, and the frequency of keywords were increased attention on the variables and determinants of frailty [27]. The trends of frailty research are clearly differentiated in the 2000s compared to previous periods, when the research topics became more refined and specialized, and when the determinants of frailty, comprehensive assessments, and measurements were the primary topics of concern. Elderly cancer patients, determinants of frailty, measurements of frailty, and research on cognitive impairments, such as dementia, are currently the main topics of frailty [28]. In the 2000s, there was an emphasis on nutrition in particular and dementia. The phenotype of frailty was also emphasized, so measurements using phenotypes were predominant. The keywords of this period included ‘homecare’, ‘nutrition’, ‘comorbidity’, ‘sarcopenia’, ‘albumin’, and ‘dementia’. In the 2010s, studies have been widely conducted, including various research on frailty indexes, assessment, and outcome variables. The comprehensive measurements have become more important than the exterior aspects of frailty [10]. Specifically, continuous studies have been conducted examining the physical, mental determinants, physical functions, and biomarkers of frailty. ‘Dementia’ was found to be the keyword with the highest degree centrality, showing that studies on cognitive function are those being most actively conducted (Fig 1).

Betweenness centrality refers to a position of a node among other nodes within a network; it is normally calculated as the fraction of the shortest paths between node pairs that pass through the node of interest. It affects the connection and flow of the entire network [29]. In other words, it is a keyword that mediates sub-topics and a concept that extends onto other topics of related research. In terms of core-keywords with high betweenness centrality, they were outcome factors such as ‘death’ in the 1980s and determinants like ‘nutrition’ and ‘aging’ in the 1990s. In the 2000s, determinants like ‘nutrition’ and ‘aging’ remained some of the top keywords followed by ‘chronic disease’ and ‘home care’. Since 2010, keywords about determinants, including ‘nutrition’ and ‘aging’, were seen at the top, and new keywords such as ‘obesity’, ‘inflammation’ and ‘biomarker’ also appeared at the top rank. In the 2010s, a huge network and an independent sub-network of care and geriatric cancer patients were created but no intermediary was observed between the two networks (Fig 2). As such, there is a need for research that can link geriatric cancer patients and care with other concepts such as nutrition, biomarkers, and geriatric assessment.

This study found that most of the keywords with high degree centrality also exhibited high betweenness centrality, thereby serving as core-keywords of high influence that represent each period. A keyword that was ranked at the top with higher centrality was named ‘core-keyword’ for its semantic connection to other keywords, and a core-keyword was considered a theme, which represents a given study. According to the results of sociogram analysis (Figs 1 and 2), frailty research in the 1980s was done in relation to death by focusing on nursing-home, in-patients, and Medicare. Frailty among patients in nursing-home and hospitals and those entitled to Medicare were the main focus of the research at that time, and frailty was analysed in relation to death. Frailty research in the 1990s frequently discussed aging, geriatrics, and long-term care, and there were also a number of studies on malnutrition, aging, and longevity. Naturally, the focus group of frailty research at that time was shifted to the elderly. The core-keywords in the 2000s presented equally increased degree centrality, meaning, and expansion of the frailty research network. It indicates that the development of research, and home care, nutrition, comorbidity, and sarcopenia were widely examined at that time. In other words, the place and target of frailty care changed from facilities to home care. Since frailty accelerates when accompanied by illness, comorbidity care for the elderly is essential, and with the emergence of sarcopenia among the key issues, there is a need for active research about the condition [30]. As interest in nutrition continued in the 2010s, malnutrition served as the key concept in frailty research. During this period, malnutrition was examined in conjunction with geriatrics, mobility, and epidemiology, and comprehensive geriatric assessment was discussed actively in line with geriatric oncology and nursing. In terms of developmental stage of research, the research has extended to gradually embrace the results of frailty, its causes, and measurement, and further research should examine how to prevent frailty.

Analysing degree centrality showed that 12 out of the top 30 ranked keywords are new in the 2010s. These are ‘geriatric oncology’, ‘general practice’, ‘obesity’, ‘comprehensive geriatric assessment’, ‘biomarker’, ‘physical performance’, ‘frailty index’, ‘longitudinal study’, ‘oxidative stress’, and ‘cognitive impairment’. This shows that research on the field of frailty is an expanding field with the emergence of new topics. The newly emerged topics examine the characteristics of subjects (geriatric oncology), determinates of frailty (biomarkers, oxidative stress, obesity), and measurements of frailty (comprehensive geriatric assessment, physical performance, frailty index). Moreover, cognitive impairment, which includes dementia, has been a newly emerging topic in this time period. As keywords on qualitative research, interventions, and preventions were not extracted, these areas should be implemented in future research.

In the 1980s and 1990s, sub-research was not fully formed in the early stages of the research, but in the 2000s the sub-research of frailty research developed. In the 2000s frailty research, sub-themes were sarcopenia, dementia and disability, indicating that frailty was investigated from the view of disease [31, 32]. In the 2010s, obesity, nutrition, prevention, evaluation, and ADL were sub-themes of the research network that focused on frailty prevention. Comparing communities of frailty research enabled us to confirm changes in periods of frailty research [33, 34, 35].

Early detection is important for the prevention of frailty, but worldwide, including in Korea, efforts to recognize frailty as a public health concern are not sufficient. It is worth noting that, this study will help understand frailty research and we looked at the trend of frailty research from a new perspective.

This study did not consider the influences of the main keywords by treating them with singular values and did not assign separate weightings to them, so this may have influenced the research findings. Additionally, the current study failed to completely remove the index effect as it selected and analysed keywords for the research data. Even though this study analysed the co-occurrences of keywords, in the future, using keyword phrases or combinations of keywords as the subject would enable a more detailed understanding of the keyword concepts. We represented our data as static networks and explored knowledge structure. However, most networks change through time. Therefore, ERGM (exponential random graphs model) will be recommended to test assumptions about the dynamic process driving this change in the future. Even though this study has limitations listed above however, this study proposed a microscopic perspective by reviewing all research on frailty published from the 1980s to the 2010s, and thus, sought to summarize the trends of research on frailty. It will contribute to suggest topics in related fields in need of further investigation.

## Conclusions

This study provides a systematic overview of frailty knowledge development. ‘Dementia’ was found to be the keyword with the highest degree centrality, showing that studies on cognitive function are those being most actively conducted in recent decade. In the 2000s frailty research, sub-themes were sarcopenia, dementia and disability, indicating that frailty was investigated from the view of disease. In the 2010s, obesity, nutrition, prevention, evaluation, and ADL were sub-themes of the research network that focused on frailty prevention.

## Supporting information

S1 Table Data characteristics. (TIF)

S1 Fig Dendrogram of community analysis. (TIF)
