# Supplementary figures and images for: Mapping the knowledge structure of frailty in journal articles by text network analysis
Source: PLoS One. 2018 Apr 19;13(4):e0196104. doi: 10.1371/journal.pone.0196104 (PMC5908161; doi:10.1371/journal.pone.0196104)

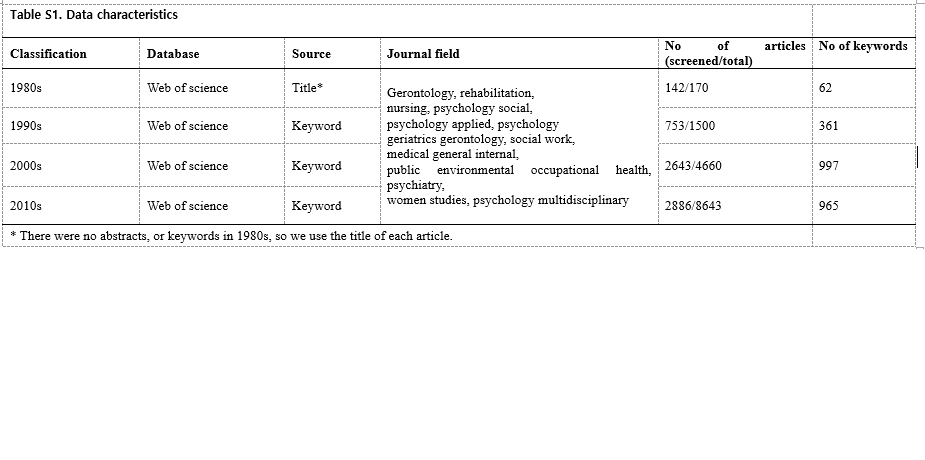

Supplement: S1 Table — (TIF) [file pone.0196104.s001.tif]

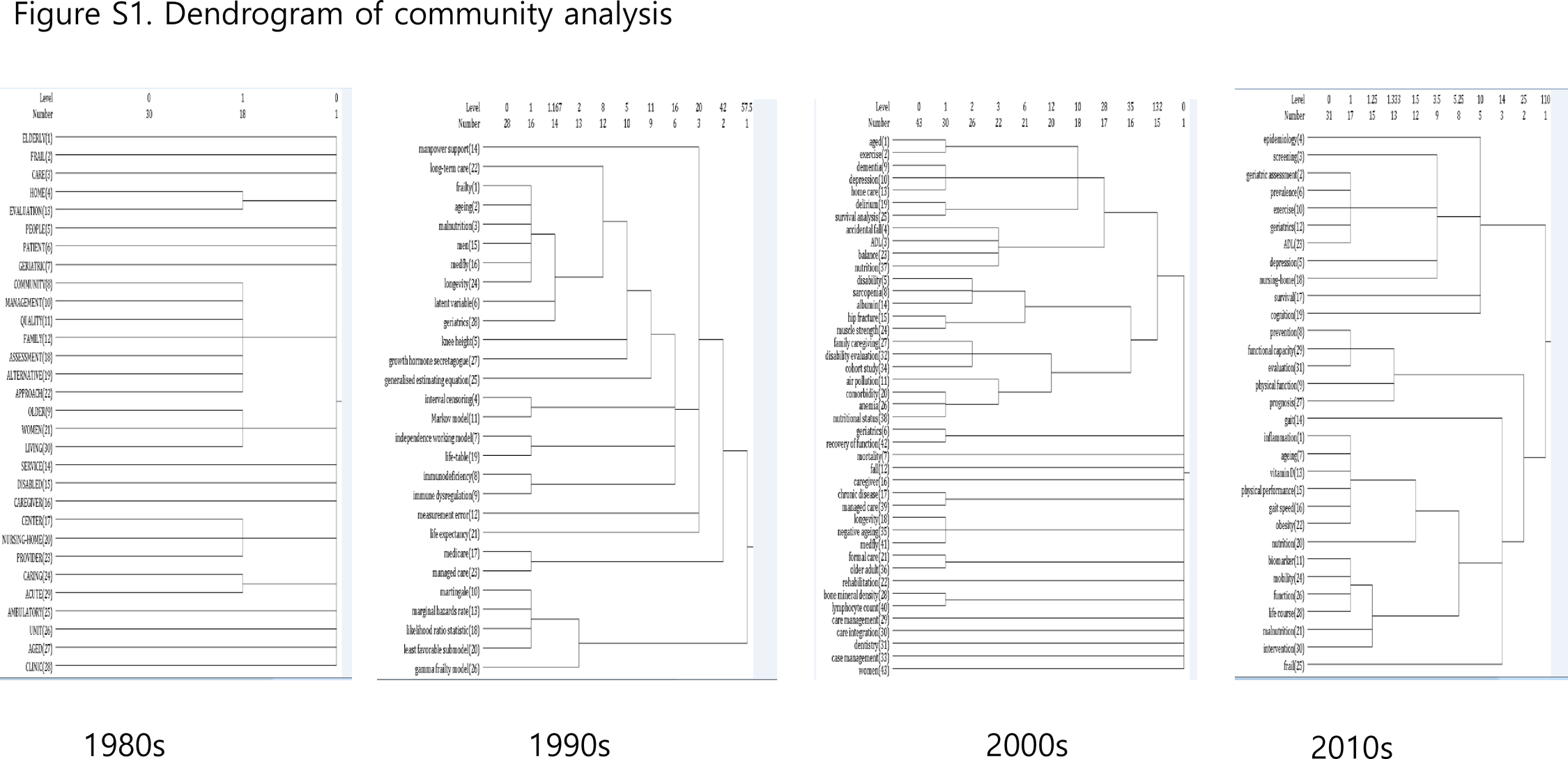

Supplement: S1 Fig — (TIF) [file pone.0196104.s002.tif]
